# Music-based and auditory-based interventions for reading difficulties: A literature review

**DOI:** 10.1016/j.heliyon.2022.e09293

**Published:** 2022-04-18

**Authors:** Alice Cancer, Alessandro Antonietti

**Affiliations:** Department of Psychology, Università Cattolica del Sacro Cuore, Milan, Italy

**Keywords:** Dyslexia, Music, Auditory training, Reading, Phonological awareness

## Abstract

Remediation of reading difficulties through music and auditory-based interventions in children with impairments in reading (such as developmental dyslexia) has been suggested in light of the putative neural and cognitive overlaps between the music and language domains. Several studies had explored the effect of music training on reading development, showing mixed results. However, to date, the meta-analyses on this topic did not differentiate the studies on typical children from those on children with reading difficulties. To draw a clear picture of the remedial effects of music-based and auditory-based interventions, the present review of the literature included studies on struggling readers only. Eighteen studies have been categorized according to the type of the main training activity – either specific auditory training or more broad music training – and the combination with reading exercises. The reviewed studies showed that musical and auditory interventions yielded a positive, but not consistent, effect on reading. Nevertheless, significantly larger improvements of phonological abilities, relative to the control conditions, were overall reported. These findings support the hypothesis of a transfer effect of musical and auditory training on phonological and literacy skills in children with reading difficulties.

## 1 Introduction

Reading difficulties are common among students in paediatric age and can potentially hinder academic learning. They can be associated with a general learning impairment, environmental problems, or a specific learning disorder in the ability of reading, namely, developmental dyslexia (DD) (American Psychiatric Association, 2013). The behavioural manifestations of reading impairments, and specifically DD, include an inaccurate and/or slow decoding of written language, resulting in a hesitant and effortful reading. Such difficulties may stem from a dysfunctional grapheme-phoneme mapping (i.e., the process of mapping print letters to the sounds of one’s language) (Snowling and Hulme, 2012). Given the multidimensional clinical manifestation of DD, several etiological theories of DD (which can be tentatively extended to general reading impairments) have been posited, among which the magnocellular theory (e.g., Stein, 2001), the visuo-spatial attention deficit hypothesis (e.g., Franceschini et al., 2012), and a multifactorial neurocognitive explanatory model (Menghini et al., 2010). In addition to that, evidence from behavioural studies showed concurrent deficiencies in phonological awareness, namely, the representation, storage, and retrieval of speech sounds (Ramus et al., 2013). The acquisition of sublexical knowledge is indeed crucial for learning letter-sound correspondence rules (i.e., phonological decoding) and, thus, for successful reading.

In the domain of reading difficulties, because of more precise identification criteria, specific conjectures about the allegedly involved mechanisms have been proposed with reference to DD rather to reading impairment defined in other ways. More precisely, neurophysiological and cognitive evidence led authors to formulate the hypothesis according to which musical and auditory training could have a positive impact on phonological and reading difficulties, by enhancing auditory temporal processing abilities, which are putatively compromised in children with DD (for a review, see Hämäläinen et al., 2013). An atypical oscillatory neural activity in the auditory cortex may contribute to the development of atypical functioning of the left-hemisphere reading network (Lallier et al., 2017; Cantiani et al., 2019), which was suggested to ultimately hinder the phonological and reading development in children with DD (Goswami et al., 2011; Giraud and Ramus, 2013). More precisely, according to the Temporal Sampling (TS) theory (Goswami, 2011; Goswami et al., 2014), such atypical neural entrainment is associated with an impairment in the perception and discrimination of amplitude envelope (AE) rise times (i.e., the rates of change of modulation onsets in the amplitude envelope) of sounds. AE rise times are temporal acoustic components carrying information about the rhythmical structure for both speech and non-speech (such as musical) sounds. Rise times are critical events in the speech signal as they underlie speech rhythm and the prosodic structure of language, thus facilitating the temporal segmentation of the acoustic signal (speech stream) into syllables and words. An accurate processing of speech rhythm is crucial for language acquisition and development (e.g., Curtin, 2010). The distribution of the stressed units in the continuous stream of phonemes acts as a phonological framework to encode lexical units and acquire new vocabulary (Leong et al., 2014), which later would contribute to reading acquisition (Ziegler et al., 2014).

Impaired auditory processing of speech cues was indeed found to be correlated to difficulties in processing non-speech rhythm in individuals with DD, as measured by musical tasks assessing the sensitivity to the metrical structure of sounds (Huss et al., 2011; Goswami et al., 2013; Flaugnacco et al., 2014). Performance in rhythm discrimination tasks was found to be a strong predictor of phonological awareness and reading development in children (Huss et al., 2011; Flaugnacco et al., 2014; Ozernov-Palchik et al., 2018), as well as a longitudinal predictor of reading development (Goswami et al., 2013; Cancer and Antonietti, 2018). Based on these premises, it was suggested that musical activities based on improving temporal synchronization, which involve a fine-grained temporal perception of the acoustic cues, would be an efficient training tool for addressing the phonological and reading difficulties characterizing DD (Overy, 2003; Tallal and Gaab, 2006; Patel, 2012).

Several studies had explored the effect of music training on reading development. However, results of such studies are mixed. To our knowledge, to date, four meta-analyses addressed the relationship between music training and literacy-related skills (Butzlaff, 2000; Standley, 2008; Cogo-Moreira et al., 2012; Gordon et al., 2015), considering different parameters and selection criteria. The first meta-analysis (Butzlaff, 2000) did not find any significant effect of music on reading skills; However, only a small number of experimental studies, and with a considerable variation of effect sizes, was included. The second meta-analysis (Standley, 2008) found a significant, moderate effect of music intervention on reading (overall effect size of Cohen’s *d* = .32) and larger effects when music activities incorporated reading tasks (*d* = .44) or when music was used as a contingency to reinforce reading (*d* = .66). Results of the most recent meta-analysis (Gordon et al., 2015) supported the hypothesis that music training leads to phonological awareness skill improvement, however with modest gains (effect size of *d* = .20). As for reading fluency, no significant transfer effect emerged (with a non-significant effect size of *d* = .16).

None of these meta-analyses differentiated the studies on typical readers from those on children with reading difficulties. Consequently, the effect of music training on reading and phonological skills was investigated in diverse subject populations considered together. We argue that the atypical skills of individuals with reading difficulties should be taken into account. More precisely, we suggest that the responses to musical and auditory training would vary substantially depending on the level of initial auditory processing and reading abilities and, as a consequence, typical readers and children with reading difficulties should be considered separately. The only two attempts to selectively analyse studies on children and adolescents with a diagnosis of DD was made by Cogo-Moreira et al. (2012) and by Rolka and Silverman (2015). The first authors planned to include in their meta-analysis only randomized controlled trials (RCTs) with at least one reading outcome, which they could not find among the initially retrieved studies. Therefore, they concluded that no evidence on the effectiveness of music for the improvement of reading skills in children and adolescents with DD was available at the time of their exploration. In the attempt to describe research on music and DD, without excluding non-RCTs studies, Rolka and Silverman (2015) reviewed 23 studies on children and adults with DD or with dyslexic traits. However, only a portion of the selected studies aimed at testing the effectiveness of music intervention, whereas other studies explored the neural correlates of music processing in DD or performances of individuals with DD in music tasks. Positive results are reported for the great majority of the intervention studies, but these results are not discussed based on the characteristics of the training methods.

## 2 Aims and methods

We aimed to conduct and report an updated review of the literature about the specific effect of music-based and auditory-based interventions for reading difficulties in children and adolescents, broadening the selection criteria. The umbrella term “reading difficulties” will be used in the review to indicate the presence of a reading performance that is significantly deviant from the norm, therefore referring to a broader condition, which includes a diagnosis or risk of DD. Furthermore, we aimed to discuss the outcomes of the interventions, based on the main component of the intervention program (i.e., auditory processing or music) and on the combination between auditory/musical activities and reading activities. We retrieved the articles from PubMed, PsycInfo, and Scopus electronic databases by using the following keywords: (‘music’ OR ‘auditory training’) AND (‘dyslexia’ OR ‘reading’). The time period was left open. We searched for additional references in the retrieved articles and checked each article according to our inclusion criteria. The article selection was based on the following criteria: (a) peer-reviewed publications written in English; (b) studies measuring the effect of training programs including an auditory and/or a music component; (c) studies on children with DD, poor reading, or another learning disability (LD) affecting reading; (d) studies including at least one of the following primary outcomes, measured using standardized tests: reading skills (i.e., reading aloud passages, words, or pseudo-words), passage comprehension, phonological awareness, spelling; (e) controlled studies (active control or no-treatment); Within-group intervention comparisons were also included; (f) inclusion of pre-vs. post-intervention comparison measures. The eighteen studies included in the review (see Table 1) have been categorized according to (i) the type of the main component of the intervention program (i.e., specific auditory processing training or music training) and (ii) the absence or presence of reading activities embedded in the auditory/musical intervention. Thus, the studies have been grouped into four categories, based on the characteristics of the tested intervention: (1) auditory processing training only, (2) auditory processing and reading training combined, (3) music training only, (4) music and reading training combined.

**Table 1:** Study characteristics. For each study, results are reported as symbols representing whether the experimental intervention group outperformed the control group (>), or both groups improved similarly (=), or none of them improved (×) immediately after training (pre–post training between-group comparisons).

Study	Language	Participants’ mean age, diagnosis	Treatment duration	Experimental intervention	Control intervention	Reading outcome measure	Reading improvement relative to control group	Secondary outcome measures	Secondary outcome improvement relative to control group
**Auditory Processing Training Only**									
Hayes et al. (2003)	English (US)	*N = 42* 9.81, learning problems	8 weeks (60 min per day, 5 days a week)	*N = 27* *Earobics*: auditory discrimination training (1:1)	*N = 15* No-treatment	Wide Range Achievement Test-3	×	Spelling	×
								Auditory processing	>
								Word memory	×
								Listening comprehension	×
McArthur et al. (2008)	English (US)	*N = 57* ≈10.6, dyslexia (*N = 20*) 10.2, typical reading (*N = 37*)	6 weeks (30 min per day, 4 days a week)	*N = 20* Rapid auditory processing and frequency discrimination training (1:1)	*N = 37* No-treatment	Castles and Coltheart’s Irregular Word List	×	Spoken language	=
								Spelling	=
								Arithmetic	×
								Visual discrimination	×
Thomson et al., 2013	English (UK)	*N = 33* 9.4, dyslexia	6 weeks (30 min sessions, once a week)	*N = 9* Rhythmic intervention training rise time discrimination (1:1)	*N = 12* *Phonomena*: phoneme discrimination training (1:1) *N =12* No-treatment	Test of Word Reading Efficiency	=	Spelling	>
								Phonological awareness	=
								Rise time discrimination	>
								Auditory duration and intensity discrimination	=
**Auditory Processing and Reading Training Combined**									
Merzenich et al. (1996); Tallal et al. (1996)	English (US)	*N = 22* 7.4, reading impairment	4 weeks (≈180 min per day, 7 days a week)	*N = 11* *Fast ForWord*: auditory processing + language training (1:1)	*N = 11* Language training (1:1)	-	-	Rate of acoustic processing	>
								Speech discrimination	>
								Language comprehension	>
Temple et al. (2003)	English (US)	*N = 32* 9.9, dyslexia (*N = 20*) 10.7, typical reading (*N = 12*)	8 weeks (100 min per day, 5 days a week)	*N = 20* *Fast ForWord*: auditory processing + language training (1:1)	*N = 12* No-treatment	Woodcock- Johnson Reading Mastery Test-Revised	>	Receptive and expressive language	>
								Rapid naming	>
								Left-temporoparietal-metabolic activity (fMRI)	>
**Music Training Only**									
Roskam (1979)	English (US)	*N = 36* ≈7.5, learning disability	12 weeks (60 min sessions, twice a week)	*N = 12* Music therapy (Group) *N = 12* Music therapy (Group) + learning disability rehabilitation (1:1)	*N = 12* Learning disability rehabilitation (1:1)	Peabody Individual Achievement Test	=	Spelling	=
								Passage comprehension	=
								Auditory discrimination	=
Douglas and Willatts (1994)	English (UK)	*N = 12* 8.9, poor reading	24 weeks	*N = 6* Music activities for improving auditory, visual and motor skills (Group)	*N = 6* Discussion skills training (Group)	Schonell Reading Test	>	-	-
Overy (2003)	English (UK)	*N = 9* 8.8, dyslexia	15 weeks (20 min sessions, 3 times per week)	*N = 9* Music lessons (Group)	*N = 9* Within-group no-treatment period	WORD Test	=	Spelling	>
								Phonological skills	>
								Rhythmic skills	>
								Auditory skills	>
Cogo-Moreira et al. (2013)	Portuguese (Brazil)	*N = 235* 9.15, poor reading	20 weeks (50 min sessions, 3 times per week)	*N = 114* Music classes (Group)	*N = 121* No-treatment	*ad hoc* word, non-word and text reading tasks	> (except for non-word reading)	Phonological assessment	>
								Portuguese achievement	>
								Math achievement	>
Bhide et al. (2013)	English (UK)	*N = 19* 6.8, poor reading	8 weeks (≈25 min sessions, ≈2 times per week)	*N = 10* Music program training rhythm perception, syllable stress, and rise time discrimination (1:1)	*N = 9* *GraphoGame Rime*: grapheme-phoneme correspondences training (1:1)	Test of Word Reading Efficiency	=	Spelling	=
								Verbal working memory	=
								Auditory processing	=
Habib et al. (2016)	French	*N = 12* ≈9.5, severe dyslexia	6 weeks (3 h per week)	*N = 12* *Cognitivo-Musical Training*: rhythmic perception multisensory training (Group)	*N = 12* Within-group no-treatment period	Khomsi test	>	Phonological awareness	>
								Verbal working memory	×
								Auditory attention	>
								Visuo-spatial attention	×
								Sequential visual processing	×
								Rhythm reproduction	×
**Music and Reading Training Combined**									
Register et al. (2007)	English (US)	*N = 41* ≈7.5, dyslexia (*N = 8*) ≈7.5, typical-reading (*N = 33*)	4 weeks (3 times per week)	*N = 25* Music program from improving reading, comprehension and vocabulary (Group)	*N = 16* No-treatment	Gates-MacGinitie Reading Test	=	Vocabulary	=
								Passage comprehension	×
Flaugnacco et al. (2015)	Italian	*N = 46* 10, dyslexia	30 weeks (60 min sessions, twice a week)	*N = 24* Music class based on Kodaly and Orff pedagogy (Group)	*N = 22* Painting class (Group)	MT Reading Test and Assessment battery for Developmental Reading and Spelling Disorders – DDE-2	> (Text and pseudo-word reading accuracy)	Phonological awareness	>
								Verbal working memory	>
								Auditory memory	=
								Temporal processing	>
								Self-esteem	=
Bonacina et al. (2015)	Italian	*N = 28* 12, dyslexia	5 weeks (30-min sessions, twice a week)	*N = 14* *Rhythmic Reading Training:* beat-based computerized reading exercises (1:1)	*N = 14* No-treatment	MT Reading Test and Assessment battery for Developmental Reading and Spelling Disorders – DDE-2	> (Pseudo-word reading speed, word and text reading accuracy)	Rhythm reproduction	×
Cancer et al. (2020)	Italian	*N = 24* 10, dyslexia	3 weeks (2 45-min session, 3 times per week)	*N = 12* *Rhythmic Reading Training:* beat-based computerized reading exercises (1:1)	*N = 12* Visual Hemisphere-Specific Stimulation + Action Video Game Training (1:1)	MT Reading Test and Assessment battery for Developmental Reading and Spelling Disorders – DDE-2 and Word and pseudo-word reading test (WPRT)	> (Pseudo-word reading accuracy)	Phonological awareness	=

## 3 Auditory processing training only

### 3.1 Earobics

The Precise Auditory Timing Hypothesis (PATH) (Tierney and Kraus, 2013, 2014; Woodruff Carr et al., 2014), according to which precise timing perception at rapid temporal rates is crucial for speech perception and phonological development, served as the framework for Hayes et al. (2003), who tested the efficacy of an auditory processing training program. The authors aimed to study the cortical representation of speech, in quiet and in noisy environments, in children with learning disorders (LDs). Electrophysiological evidence showed anomalies in the cortical responses to speech syllables in quiet (Cunningham et al., 2000) and noise (Cunningham et al., 2001; Wible et al., 2002) environments in learning impaired children. To study the impact of auditory perceptual training on cortical responses, learning achievement, and auditory processing skills, a group of 42 children with learning problems (LP) – including attention deficit disorder and/or reading impairment and/or spelling impairment – was enrolled in a test-training-retest study. Twenty-seven children received an intervention using *Earobics* (Cognitive Concepts Inc., Evanston, IL) (Diehl, 1999), a commercial computerized auditory training program consisting of audio-visual exercises in quiet and noise conditions. More precisely, the activities included phoneme discrimination, auditory memory, auditory sequencing, auditory attention, rhyming, and sound blending skills. The experimental group (N = 27) participated in 35–40 training sessions of 60 min, over an 8-week period, whereas the control group (N = 15) did not receive any intervention. Reading, spelling, auditory processing skills, word memory, and listening comprehension, along with cortical potentials, were tested 8 weeks apart. Results showed a significant improvement of auditory processing in the trained group, along with latency and amplitude decreases in cortical potentials in quiet, and more robust cortical responses in noise conditions, relative to the untrained group. However, no improvement of the other behavioural measures (i.e., spelling, word memory, and listening comprehension) was found in either group.

### 3.2 Rapid auditory processing (RAP) training

McArthur et al. (2008) pointed out that most studies inspired by the Rapid Auditory Processing (RAP) approach, which posited the association between the processing of the rapid spectro-temporal characteristics of phonemes and the acquisition of phonological representations of spoken words (Tallal et al., 1993; Tallal, 2004; Tallal and Gaab, 2006), trained non-speech auditory processing in conjunction with other complex linguistic skills. According to the authors, such a procedure hides the specific impact of auditory processing training on reading and language improvement. To address the specific effect of RAP, frequency discrimination, consonant-vowel discrimination, and vowel discrimination training on reading and language skills, the authors tested and trained 20 children with DD and 8 children with specific language impairment (SLI), aged 6–15. Participants’ reading, spoken language, spelling, arithmetic, and visual discrimination abilities were assessed pre-, post-, and 6 weeks after an auditory intervention. Auditory temporal processing skills (i.e., frequency discrimination, rapid auditory processing, vowel discrimination, consonant-vowel discrimination, auditory sustained attention) were also tested using validated psychoacoustic tasks. Participants were trained for 30 min per day, 4 days per week, for 6 weeks using a computerized program that included a game version of the same psychoacoustic tasks used in the assessment, by adaptively lowering discrimination thresholds. A control group of untrained typical readers (N = 37) was tested 6 weeks apart. Pre- vs. post-intervention differences in the outcome measures were measured only for the subgroup of trained participants (25 out of 28) whose scores in the psychoacoustic tasks were in the normal range after the training. Results revealed that a successful RAP training led to significant improvements in spelling and spoken language (namely, a sentence repetition task), but not reading, in both children with DD and SLI. However, test-retest gains of the untrained typical readers were similar to those observed in the trained group. Therefore, the authors concluded that the improvements observed in children with DD and SLI after the intervention was not a genuine effect of sound training, and thus RAP and speech discrimination training was ineffective.

### 3.3 A rhythmic intervention for rise time perception

Thomson et al. (2012) compared the efficacy of two auditory processing interventions for DD, namely, (a) a novel rhythm-based auditory training program and (b) a commercial computerized program aimed at training the phonemic contrast discrimination, called *Phonomena*. The theoretical underpinning for the rhythm-based training was the TS theory (Goswami, 2011; Goswami et al., 2014), according to which impaired phonological processing in DD is related to an impairment in the perception of the AE rise times (i.e., the rates of change of modulation onsets in the amplitude envelope of a sound wave). An adaptive intervention was devised to target the rhythmic perceptual difficulties putatively associated with rise time deficits in DD (Thomson and Goswami, 2008), by training both the rise time discrimination and the perception of metrical stress patterns in speech and non-speech rhythms. The rhythmic training comprised music activities (e.g., rhythm copying and rhythm synchronization on djembe drums) and computer-based games, namely, an adaptive amplitude rise time discrimination task, the prosodic *Dee-Dee* game (a name-matching game in which the target name is replaced by the reiterative syllable /*dee*/, so to eliminate distinctive phonetic information while retaining the stress, rhythm, and intonation of the original name) (see Goswami et al., 2010), and an amplitude rise time synchrony game. As for the phoneme-based intervention, it had been previously shown to yield significant word discrimination and phonological awareness improvements in 8–10 years old children after a 4-week intervention, relative to regular classroom activities. Thirty-three children with DD (mean age =9.4 years) were assigned to one of three conditions: the rhythmic training, the phonemic training, or a no-treatment control group. Both experimental groups participated in a 6-week training program, comprising 30-minute, weekly, one-on-one sessions. Participants’ reading, expressive vocabulary, spelling, phonological awareness, phonological short-term memory, rapid automatized naming (RAN), and auditory processing (i.e., amplitude rise time, duration, and intensity discrimination) were tested 6 weeks apart. Both the rhythmic intervention and the phonemic intervention yielded a significant improvement of phonological awareness, relative to the untrained control group. Moreover, the rhythmic intervention had a significant positive effect on rise time discrimination and spelling. However, neither intervention was effective in improving reading. The authors argued that, since children were trained for a relatively short time (6 weeks), a longer intervention, together with a larger sample size, could have led to further significant results.

## 4 Auditory processing and reading training combined

### 4.1 Fast ForWord (FFW) acoustic training program

Tallal and colleagues (Tallal et al., 1993; Tallal, 2004) identified a low-level auditory processing impairment as the basic cause of dyslexia-related phonological deficits. Individuals with DD often show deficiencies in temporal resolution of rapidly changing auditory stimuli that may, in turn, influence speech perception (Tallal et al., 1993; Tallal, 2004; Tallal and Gaab, 2006). Accordingly, an auditory-based remediation program for DD, designed to improve both dynamic auditory and phonological processing skills, called *Fast ForWord* (FFW) (Scientific Learning Corporation, Oakland, CA), was devised by the authors (Merzenich et al., 1996; Tallal et al., 1996; Tallal, 2004). FFW is an adaptive neuroplasticity-based computerized training program, which incorporates two approaches to intervention disguised as computer games. The first approach consists of an auditory tone sequencing exercise (*Circus sequence*) aimed at speeding up rapid auditory processing and expanding the memory span for rapidly successive events. More precisely, the subject has to indicate the temporal order of tones that either rise or fall in pitch and that feature the frequency range and speed that typify the temporo-spectral changes occurring in formant transitions in consonants. The second approach comprises a series of language-based exercises aimed at training individual components of language and reading across multiple levels, from phoneme (*Phonic Match*) to word (*Phonic Word*), to phrases (*Language Comprehension Builder*). In all language exercises, auditory speech streams are acoustically modified by a computer algorithm, so as to amplify the rapidly successive acoustic changes naturally occurring in the ongoing waveform of speech, and thus emphasizing the differences between brief phonemes. In a first trial using FFW (Merzenich et al., 1996), 7 learning-impaired children with reading deficits, aged 5 to 10, participated in an intensive 4-week intervention, during which they trained 3 h per day, 5 days a week at the laboratory, plus 1–2 h per day, 7 days a week at home. Speech, language, and auditory temporal processing abilities were assessed before and after training using a battery of standardized tests. Within-group post-training performances in speech discrimination and language comprehension significantly improved after training.

In a second trial including 22 reading-impaired children aged 5–10, two subgroups of participants received for 4 weeks either FFW training or a control treatment, which comprised the same language intervention but with unmodified speech and non-temporally adapted visual computer games. After training, the experimental group showed a significantly greater improvement in the rate of acoustic processing, compared to the control group (Merzenich et al., 1996). Furthermore, significantly larger improvements were achieved by children who received FFW in speech discrimination and language comprehension (Tallal et al., 1996). Consistent with Tallal and colleagues’ hypothesis, a highly significant correlation between acoustic processing and language skills improvements emerged in the experimental group.

In the attempt to provide evidence for a transfer effect of auditory temporal processing training to reading and spelling of children with DD, Strehlow et al. (2006) implemented a training similar to the procedures described in the studies by Merzenich et al. (1996). The authors were interested in testing whether a successful training of auditory temporal processing could impact reading and spelling ability when combined with standard school-based reading training for children with DD. Forty-four German first- and second-graders with DD were assigned to one of three conditions: (a) sound processing and reading training, (b) phoneme processing and reading training, or (c) reading training only. Participants were attending a 12-week school training program for DD, for 2 h, 5 days a week. The sound (a) and phoneme (b) groups received an additional 4-week adaptive training program targeting, respectively, temporal processing of sounds and temporal processing of phonemes, whereas the control group (c) did not receive any additional intervention. Reading, spelling, sound processing, and phoneme processing of all children were tested pre-, post-, and 6 months after intervention. Furthermore, 27 participants (9 from each group) took part in a 12-month follow-up assessment. As predictable, significant improvements immediately after training were observed for sound processing in the sound group and phoneme processing in the phoneme group, relative to the other conditions. However, these effects were not as strong at 6- and 12-month follow-up testing. Spelling improvement was superior at 6-month follow-up in the sound group, but no longer at 12-month follow-up. As for reading, all groups improved significantly over time, but no difference between groups emerged. Most likely, reading gains stemmed from the school training program and auditory processing training did not contribute.

To add neurobiological evidence to FFW efficacy, two neuroimaging studies have been conducted. The first fMRI study (Temple et al., 2003) investigated the impact of FFW on both behavioural measures, including reading, and metabolic activity changes in the left temporoparietal regions. Such regions are characterized by disrupted neural responses during phonological processing in individuals with DD (for a review, see Eckert, 2004), including weakened connectivity between frontal and left temporal-parietal areas. Twenty 8- to 12-years-old children with DD participated in an 8-week (100 min per day, 5 days a week) remediation program using FFW whereas 12 untrained typical-readers served as the control group. Both groups took part in two behavioural testing sessions assessing reading, rapid naming, receptive and expressive language, 8 weeks apart. Furthermore, all participants underwent two fMRI scans while performing a phonological processing task, a non-phonological letter task, and a non-letter task. The auditory training yielded beneficial effects on reading performance (namely, word and pseudo-word reading), passage comprehension, oral language ability, and rapid naming in children with DD, together with an increase of the metabolic activity in left temporoparietal regions. However, the locus of such increased activity was near but not the same as the focus of activation observed in controls during the phonological task, which reflected, according to the authors, just a partial enhancement of the hypoactive temporo-parietal response characterizing DD. As predictable, any behavioural measure improved in untreated typical readers.

The second fMRI study (Gaab et al., 2007) investigated, at both behavioural and metabolic levels, the efficacy of FFW program. Twenty-two children with DD and 12 typical readers underwent both fMRI scan and behavioural testing assessing reading, rapid naming, listening comprehension, phonological awareness, phonological memory, and receptive and expressive language skills. Similar to the study by Temple et al. (2003), children with DD participated in an intensive 8-week intervention program, whereas control typical-readers were tested again after a period of the same length. fMRI was performed while children listened to non-linguistic acoustic stimuli designed to mimic the spectro-temporal structure of consonant-vowel-consonant speech syllables, with either rapid or slow frequency transitions. Another study performing the same protocol (Temple et al., 2000) had previously shown that, whereas in typical-reading adults the left prefrontal cortex exhibited activation, adults with DD showed no differential left prefrontal response to rapid, relative to slow, transitions. The authors found a similar left prefrontal disrupted response in children with DD, which was also partially ameliorated after remediation. In addition, all language and reading performances improved in children with DD after FFW intervention.

### 4.2 The audio-visual training of voicing

Magnan and Ecalle (2006) explored the possibility to improve reading skills in French students with DD using an audio-visual intervention designed to enhance phonological awareness. The authors argued that auditory processing training alone, in the form of segmentation of speech feedback in sublexical units (i.e., phonemes or syllables), would improve phonological awareness, but would not yield a translational effect on reading, unless the phonological stimulation was linked to the corresponding graphemes. Accordingly, they studied the efficacy of a remedial intervention focusing on grapheme-phoneme mappings, extracted from an already existing French computerized program called *Play-On* (Danon-Boileau and Barbier, 2002). More precisely, the intervention aimed at training the acoustic discrimination between pairs of consonant sounds sharing similar spectro-temporal features (i.e., /p/-/b/;/t/-/d/;/k/-/g/;/f/-/v/;/s/-/z/et/ch/-/j/), while consolidating phoneme-grapheme mappings. Participants were asked to associate the syllabic sound to the corresponding orthographic form, choosing between two printed options. Fourteen French primary school students with DD participated in a 5-week intervention (two 15-minute sessions per day, 4 days a week) delivered at school. A subgroup of participants was put on a waiting-list, so to serve as the control group, and was tested 5 weeks apart before receiving the intervention. The outcome measure was phonological awareness, whereas reading performance was not investigated. The first trained group improved in phonological awareness after intervention; However, the improvement did not differ from that of the untrained control group. Since the study was conducted in a special school for children with LDs, participants of both experimental and control groups received a phonological awareness training delivered by a speech therapist as part of the class curriculum. Most likely, this parallel standard training had a role in the phonological improvement observed in both conditions.

## 5 Music training only

A very early attempt to evaluate the effect of music training as a remedial tool for literacy difficulties was made by Roskam (1979). The experimenter designed a music therapy program aimed at training verbal and nonverbal auditory perception via group activities focused on the discrimination of duration, intensity, rhythm, pitch, and tempo. Thirty-six primary school students with LD aged 6 to 9 were assigned to one of three interventions: (a) music therapy only (N = 12); (b) music therapy combined with prescribed LDs remedial activities (i.e., writing, spelling, dictionary practice, vocabulary building, memory improvement, and visual perception training) (N = 12); (c) LD remedial activities only (N = 12). All three groups participated in a 3-month intervention comprising biweekly 60-minute sessions. Participants’ reading, spelling, passage comprehension, and auditory discrimination were assessed before and after training. Although the music therapy only group showed the highest mean gains, no significant effects of music therapy were found and all groups showed similar improvement in the outcome measures.

Another early study by Douglas and Willatts (1994) engaged 12 primary school students aged 8 to 10, who had been assessed to be poor readers by an initial reading test, in an intervention study aimed at investigating the effect of musical training on reading difficulties. Two groups matched for the initial level of reading ability participated for 6 months in either a music program or a control intervention consisting of non-musical activities designed to develop discussion skills (e.g., share ideas about a topic which was under discussion and offer individual opinions about issues which arose). The music intervention comprised rhythmic and pitch activities, devised to develop auditory, visual, and motor skills, using singing and percussions. Results showed that participants who took part in the music program significantly improved their reading relative to controls, whose post-training reading abilities were unaltered.

In a within-group intervention study, Overy (2003) tested the effectiveness of group music lessons designed for improving basic timing and auditory skills. The content of the lessons was based on singing, chanting, clapping, and percussion games combined with motor and visual activities, resulting in a multisensory intervention. Nine children with DD (mean age = 8.8) participated in the aforementioned music intervention for 15 weeks, 3 times per week (20-minute sessions). Reading abilities, together with spelling, phonological, rhythmic, and auditory skills, were assessed before and after the end of the intervention. Improvements in the outcome measures were then compared with the normal rate of development over a no-treatment period of the same length of the intervention, measured within-group. No reading improvement occurred after the music intervention, relative to spontaneous development, whereas all other outcome measures (i.e., spelling, phonological, rhythmic, and auditory skills) were significantly enhanced. The author speculated that reading improvements would have occurred if the music intervention was longer and/or more intensive.

After that, the systematic review by Cogo-Moreira et al. (2012) revealed the absence of randomized controlled trials (RCT) on music and DD, the same authors (2013) used an RCT to address the potential beneficial effect of music education classes on academic achievement of children with reading difficulties. Two hundred and thirty-five primary school Brazilian students with reading difficulties, aged 8 to 10, were assigned to either a music education program (N = 114) or a no-treatment control group (N = 121). Participants in the intervention condition took part in a 5-month music education program, 3 times per week (50-minute sessions). The group music activities focused on musical improvisation, composition, and interpretation, by encouraging students to create their own music by discovering musical elements (rhythm, melody, harmony). Participants’ word, non-word, and text reading, phonological awareness, and academic achievement on Portuguese and math grades were assessed 5 months apart. After training, all outcome measures, except non-word reading, improved significantly in the music group, compared to controls. Such results, yielded from a methodologically rigorous experimental design, corroborated the theoretical rationale behind music-based interventions for DD.

Bhide et al. (2013), adopting the TS framework for DD (for a summary, see Goswami, 2011), designed a musical intervention aimed at training the components of rhythm perception impaired in DD, together with syllable stress and rise time discrimination, via musical games. More precisely, the music intervention included the following activities: tapping a space bar at the same time as a metronome, same-different judgments on metronome tempos and short rhythms, mimicking a short rhythm, rise time discrimination, clapping and marching to the beat of a song, chanting and hand-clap games, listening to a poem and answering questions about its rhythm, and the *Dee-Dee* game (see Goswami et al., 2010). The authors investigated the impact of this musical training on verbal working memory, spelling, and auditory processing (i.e., auditory thresholds for sound rise time, duration, frequency, and intensity) of a group of 10 children with DD. Furthermore, the music training effect was compared with that of a computer-assisted intervention focusing on reading and phonological skills only, namely, the *GraphoGame Rime* (GGR). GGR is the English version of the Finnish *GraphoGame* program, which had been shown to lead to significant gains in reading (Saine et al., 2011). English GGR was previously found to be effective for 6–7 years old poor readers on spelling, non-word reading, rhyme, and phoneme awareness, relative to an untreated control group (Kyle et al., 2013). Nineteen children aged 6–7 participated in either the musical intervention (N = 10) or GGR (N = 9) for 19, 25-minute, individual sessions over a period of approximately two months. Both groups showed comparable significant improvements at post-test in all outcome measures, except for verbal working memory and rise time discrimination, in which the musical group outperformed the GGR group. Results suggested that a rhythmic training, including also activities linking metrical structure in music and language, had a comparable effect on literacy and phonological skills in struggling readers to that of a specific reading training.

Habib et al. (2016) devised a musical intervention, called *Cognitivo-Musical Training* (CMT), which incorporated several activities addressing rhythmic perception and reproduction, along with other features of the musical auditory signal (i.e., pitch, duration, tempo, pulsation). Activities were designed to engage both sensory (i.e., visual and auditory) and motor modalities (e.g., via tapping games). A group of 12 French primary school students with severe DD (7–12 years old), attending a special classroom for children with DD, participated in 18 h of group music training using CMT over 6 weeks (3 h per week). All participants were assessed four times, so to measure (a) the spontaneous improvement over a no-treatment 6-week period prior to intervention, (b) the immediate effect of the intervention, and (c) the medium-term effect of the intervention (i.e., 6-week follow-up). The assessment battery included reading, phonological awareness (i.e., phonemic blending and pseudo-word repetition), verbal working memory (digit repetition), auditory attention, visuo-spatial attention, sequential visual processing, writing, and rhythm reproduction tests. CMT yielded significant improvements in reading, phonological awareness, and auditory attention, relative to the pre-training untrained period, which persisted 6 weeks after training. No significant improvements in verbal working memory and rhythm reproduction were observed, and neither in the control variables (e.g., visual processing and writing), which were not the target of the treatment.

## 6 Music and reading training combined

Register et al. (2007) devised an intensive music program, namely *The Register Music/Reading curriculum*, specifically designed to improve word reading, passage comprehension, and vocabulary. The training sessions, occurring three times per week for 4 weeks, included music activities (i.e., music listening, singing, instrument playing, and movement) performed in combination with reading and language-based tasks, with the aid of visual stimuli and print academic material. To evaluate the effectiveness of the aforementioned instructional program, 8 second-grade students with a specific reading impairment, together with 33 typical-reading peers, were assigned to either a treatment condition (N = 25), comprising the music-reading program in addition to the curricular reading program, or a control condition (N = 16), consisting in the curricular reading program alone. All students with DD participated in the treatment condition. Reading, passage comprehension, and vocabulary skills were assessed pre- and post-intervention using a standardized battery of tests. After intervention, students with DD improved significantly in all three outcome measures. Both treatment and control classes improved significantly in reading and vocabulary, whereas none of them improved in passage comprehension. However, vocabulary gains were significantly higher in the intervention group than in the control group. Therefore, considering both DD and typical-reading students, reading was not affected by the music-reading curriculum supplement to curricular activity. Nevertheless, the specific effect of the program on students with DD is not clear, because of the lack of a matched control group.

An RCT carried out in the Italian setting by Flaugnacco et al. (2015) investigated the effect of music training, combined with homework reading activities, on phonological awareness and reading skills of children with DD. The music intervention was adapted from the Kodály and Orff pedagogic approaches to focus on rhythm and temporal processing. More precisely, music activities included the use of percussions, rhythmic body movements, sensorimotor synchronization, and chanting of rhythmic syllables. A painting training program, emphasizing visual-spatial and manual skills along with creativity, was selected as the active control condition. Forty-six Italian primary school students with DD, between the age of 8 and 10 years, participated in either music classes (N = 24) or painting classes (N = 22). Both interventions were delivered in small groups of 5–6 children each, for approximately 7 months, in 60-minute biweekly sessions. All participants received an additional standard rehabilitation program (namely, reading exercises), carried out for 20 min daily, at home, under parents’ supervision. Before and after intervention, participants were administered a battery of tests assessing reading abilities (i.e., word, pseudo-word, and text reading), phonological awareness, verbal working memory, auditory attention, temporal processing (i.e., rhythm reproduction, tapping musical meter discrimination, rise time threshold, temporal anisochrony threshold), and self-esteem. Results showed a significant improvement in the global reading outcome in both groups. However, the music group was significantly more effective in improving selectively pseudo-word accuracy and text accuracy. As for the other outcome measures, a significantly larger improvement of phonological awareness (i.e., pseudo-words repetition and phonemic blending), working memory (i.e., backward digit span), auditory attention, rhythm reproduction, and temporal anisochrony (i.e., the ability to discriminate irregular ‘jumps’ in a sequence of sounds) was observed in the music group, relative to the painting group, which, in turn, showed a larger improvement in perceptual visuo-spatial reasoning. No difference in self-esteem after training emerged between groups.

In the same year, another Italian study (Bonacina et al., 2015) tested the efficacy of a rhythm-based computerized program, called *Rhythmic Reading Training* (RRT), to improve reading in students with DD (Cancer et al., 2016, 2019, 2021, 2022; Cancer and Antonietti, 2017). The program included beat-based reading exercises, in which children would synchronize their reading to an isochronous beat. According to the authors, this approach could help the child to segment the acoustic rhythm structures in language that map to phonological units, by stressing syllables and onset-rimes, therefore maximizing the effect of the reading intervention. RRT reading exercises were designed to address diverse reading subprocesses, such as syllabic blending, sublexical reading, and lexical reading. In a first study (Bonacina et al., 2015), 28 junior high students with a diagnosis of DD, aged from 11 to 14, were split into two matching groups and assigned to either a training or a no-intervention condition. The experimental group received RRT in 9 biweekly training sessions of 30 min. The sessions were individual and supervised by a specialized trainer. Participants’ reading performances were assessed before and after the training period using a battery of Italian standardized reading tests, which provide accuracy and speed scores for reading aloud text and lists of words and pseudo-words. Rhythm perception was also assessed using a rhythm reproduction task. Pre-post changes through RRT were compared to those of the no-intervention control group. Results showed that reading abilities of participants who received RRT were significantly improved, relative to controls. More precisely, statistically significant differences were found in short and long pseudo-words reading speed, high frequency long words reading accuracy, and text reading accuracy. These results suggested that RRT induced greater changes in reading skills of students with DD relative to the natural development of reading efficiency.

A second study by the same authors (Cancer et al., 2020) compared the effectiveness of RRT to that of an intervention resulting from the combination of two visual-based interventions for DD, namely, Bakker’s Visual Hemisphere-Specific Stimulation (VHSS) (Lorusso et al., 2006) and the Action Video Game Training (AVG) (Franceschini et al., 2013). Twenty-four students with a diagnosis of DD aged 8–14 received either RRT (N=12) or VHSS + AVG (N=12) in 18 individual sessions of 45 min over the course of 3 weeks (for a total of 13 h of intervention). Results showed that both interventions significantly improved reading and phonological awareness of participants. However, RRT was found to be more effective for improvement of pseudo-word reading speed. Furthermore, this effect was found to be associated with phonological awareness improvement, as showed by correlation patterns between reading and phonology improvement measures. Such associations revealed a supporting role of phonological processes in RRT’s effect on reading.

## 7 Discussion and conclusions

Within the theoretical framework that linked the DD-related phonological deficit to anomalies in the auditory perception of temporal components of sounds (Goswami, 2011; Goswami et al., 2014), a line of research suggested that remedial methods based on improving temporal skills and increasing auditory sensitivity might be beneficial for improving the poor performances characterizing reading difficulties. Accordingly, it was proposed that musical activities, specifically based on improving temporal synchronization, would be an efficient training tool for achieving such outcome. These intervention methods have the potential to overcome the limitations of standard training methods for reading difficulties, including DD, such as expensiveness, time commitment, the tediousness of the activities, and long practice required for trainers to master the methodology. However, results on the effect of auditory and music training on phonology and reading in paediatric age, which were published in the last few decades, are mixed. We argue that the inconsistency of previous results may be attributed to the inclusion of both typical and impaired readers in the investigated samples. The present literature review aimed at discussing the specific effect of auditory and musical training on children with reading difficulties.

The results discussed in the present review showed that the most effective music/auditory approaches to the treatment of reading difficulties, considering reading and phonological processing as primary outcomes, are music training and both auditory and music interventions combined with reading activities (see Table 1). On the contrary, the mere training of auditory processing abilities was specifically effective in improving sound analysis and discrimination abilities; However, no significant transfer effects on reading and phonological awareness have been reported after auditory intervention only. We argue that music stimulates a more efficient neural encoding of rhythmical structure of sounds (Patel, 2003) through the enhancement of auditory-motor timing skills. Temporal synchronization and beat processing are better enhanced by rich and complex acoustic stimuli, such as music pieces, as showed by more efficient sensory encoding of auditory information in musicians, relative to non-musicians (Musacchia et al., 2007; Strait et al., 2014). However, the stimulation of other acoustic parameters, along with rhythm, which are typically involved in music training (e.g., pitch, duration, intensity, timbre), does not allow to isolate the specific role of rhythm. On the other hand, music/acoustic exercises, combined with a specific training of grapheme-phoneme mappings, produced significant effects on reading and phonological processing, compared to active control conditions (Flaugnacco et al., 2015; Cancer et al., 2020). Based on such evidence, the most effective music/acoustic rehabilitation approach seems to be the one in which decoding exercises are embedded in rhythm processing activities.

The heterogeneity of the reported studies, with respect to participants’ characteristics, primary outcome measures, language, type of intervention, training duration, level and type of reading difficulties, and control condition design could account for the variability in the effectiveness of interventions, and therefore should be addressed by further considerations.

Regarding our article search method, we decided to leave the time period open. As a consequence, we considered studies published within the last fifty years. The decision to include literature from such a broad time period was made to conduct a comprehensive review of a specific topic, that was not investigated before considering specific inclusion criteria on sample selection. Furthermore, while researchers’ interest in the cognitive effects of music is certainly not new (Sergeant and Vhatcher, 1974), updated functional neuroanatomical models of DD (Richlan, 2014), more recently, furthered the research on the auditory processing deficit observed in children with DD and on possible rehabilitation methods tapping such impairment specifically.

All reported studies engaged populations of children with reading difficulties. However, the selection criteria used for identifying these children differed between studies. The criteria which were applied are: (a) children with a diagnosis of DD made by independent clinicians prior to intervention; (b) children whose performance corresponded to one standard deviation below the mean (namely, poor readers); (c) children showing a failure to progress in reading at the expected level for their peer group; (d) children showing a discrepancy of at least one standard deviation between measures of intellectual functioning and reading ability (all studies excluded children with a general IQ < 85).

Concerning outcome measures, not all studies included a measure of reading performance, and those which did use tests measuring selectively one or few reading parameters (i.e., speed, accuracy, or fluency) and type of decoding (i.e., word reading, pseudo-word reading, or text reading). To better address the impact of different types of interventions on the reading process, it would be useful to have a comprehensive measure of the reading abilities.

Sharing clearly defined and rigorous diagnostic criteria and methods among researchers studying interventions for reading difficulties would help overcome these limitations. Good practices to select DD participants and provide a fine-grained assessment of their reading performance should be shared internationally. Diagnostic procedures should include a separate assessment of both reading accuracy (e.g., number of reading errors) and reading speed (e.g., syllables read per second) in multiple standardized age-normed tests (e.g., reading aloud text, words, and pseudo-words).

The heterogeneity of studies is also accounted for by language differences (i.e., English, French, German, Italian, and Portuguese). Behavioural manifestations of DD, together with the estimated prevalence in school-population, vary across languages, due to differences in orthographic depth (Snowling and Hulme, 2012; Richlan, 2014). Although dyslexia-related auditory processing deficits have been reported in both deep (Forgeard et al., 2008) and shallow orthographies (e.g., Flaugnacco et al., 2014), language differences become crucial when the auditory remedial intervention incorporates language-based activities, due also to their specific prosodic structure.

As regards training procedures, the studies have been separated into different categories according to the main component of the intervention (i.e., specific auditory processing or music training). Such categorization was made considering the nature of the main component in each training activity; However, it is worth addressing that the auditory processing interventions could include phonemic awareness or music parameter analysis training. Furthermore, the type of activities included differed greatly among studies within the same category. Auditory-based programs trained a variety of perceptual abilities, such as frequency discrimination, consonant-vowel discrimination, vowel discrimination, rise time discrimination, metrical stress patterns perception in speech and non-speech rhythms, phoneme discrimination, auditory sequencing, sound blending, etc., whereas music-based programs included disparate activities (e.g., singing, chanting, clapping games, percussion games, marching to the beat, rhythmic body movements) aimed at training different auditory skills, such as rhythm perception, rhythm reproduction, and rhythm synchronization.

Moreover, it is important to differentiate between group interventions and one-on-one interventions. All auditory-based interventions have been delivered via one-on-one sessions, whereas most music-based interventions via group classes, most likely on account of contents and tools used (i.e., computer-based training programs vs. musical instruments, singing and body movements). Therefore, based on the reported results, we cannot draw conclusions regarding the specific effect of group vs. one-on-one interventions on the outcome measures. However, we argue that the interpersonal factor could influence the treatment outcome. On the one hand, one-on-one sessions provide a better focus on individual features. On the other hand, group activities boost motivation, compliance, and enjoyment.

No clear evidence of the influence of training duration on intervention efficacy can be derived from the present collection of experimental studies. Significant effects on reading have been reported both after short (4 weeks, e.g., Habib et al., 2016) and long interventions (30 weeks, e.g., Flaugnacco et al., 2015), regardless of intensiveness.

Finally, a methodological consideration should address the differences in the design of the control group condition. To control for test-retest effects, for spontaneous reading development, and for the influence of other confounding factors, a no-treatment control group should be used. However, to control for Hawthorne effects, an active control condition should be included. Only one of the presented studies included both control conditions (Thomson et al., 2012). Active control conditions included: standard LD rehabilitation, specific reading training, visual-based training, language training, phoneme discrimination training, discussion skills training, or painting class. In most studies, a group of atypical readers matched for the relevant variables (e.g., age, gender, IQ, baseline performances) was assigned to the control condition (active or no-treatment), whereas in few studies untrained typical-readers served as the control group. It should be noted that Temple and colleagues’ (2003) and Register and colleagues’ (2007) results should be considered with caution, given the unmatched control group of typical readers. Furthermore, two studies included a within-group condition, in the form of a no-treatment period prior to intervention. Positive outcomes have been observed in both active-controlled studies and in studies with a no-intervention control condition. In studies where the auditory/musical intervention was delivered in conjunction with reading or language-based training activities, the comparison with an untrained group cannot clearly differentiate the effect accounted for by the musical/auditory component from that accounted for by the linguistic component. Conversely, if both experimental and active control treatments shared a similar reading or language-based training, the additional effect of music/auditory training could thus be measured.

Overall, the collection of studies presented in this review showed that musical and auditory interventions, designed to address the DD-related phonological deficits, yielded a beneficial effect on reading to some extent. The comparison between musical/auditory training programs and standard, language-based only, remediation approaches revealed – in most cases – comparable efficacy. Furthermore, all the studies which included phonological awareness as the primary outcome showed significantly larger improvements of phonological abilities, relative to control conditions (except for Thomson et al., 2012). In addition, auditory processing skills were also enhanced by most interventions considered in this review. Therefore, such findings support the hypothesis of a transfer effect of musical/auditory training on phonological skills.

The hypothesis of a transfer effect of enhanced music perception skills to the language domain is supported by relevant experimental (e.g., Schön et al., 2004) and theoretical work (e.g., Besson et al., 2011). According to the model proposed by Overy (2003) and later modified by Tallal and Gaab (2006), musical training would specifically improve the processing of rapid temporal acoustic cues. A functional analysis of the temporal components of speech sound is crucial for the acquisition of phonological representations of words and phoneme-grapheme mappings, which are the basis of reading development. However, in some of the reviewed studies, improvements in phonological and auditory processing produced by the interventions failed to yield also improvements in reading. It may be that the contribution of phonological and auditory skills to reading varies depending on the kind and level of reading impairment of each participant. We suggest that in case of non-phonologically-based reading problems, other impaired processes (e.g., visuo-attentional), which had not been stimulated by the musical/auditory training, may prevent the improved phonological and auditory processes to impact on reading. Although currently there is no consensus about a classification of reading problems subtypes, differences in clinical manifestations can be interpreted as results of specific impaired subprocesses. In particular, a word reading deficit can stem from a specific dysfunction of the lexical route of reading (Coltheart et al., 2001), which is based on visual word recognition processes; Whereas a pseudo-word reading deficit can be better attributed to an impairment of the phonological route of reading (Coltheart et al., 1993) and grapheme-phoneme mappings. We speculate that differential reading profiles can benefit most from an intervention addressing each specific impaired subprocess, and specifically readers who show deficits in phonological processes should take benefit to the best from musical/auditory interventions. Future research should explore this hypothesis by studying the specific contribution of music/auditory interventions on specific subtypes of readers.

Another possible hypothesis is that, in order to observe the music/auditory training’s cascading effect on reading skills, long-term effects should be consider. We suggest that future research should include the use of longitudinal studies to test possible delayed reading effects, stemming from enhanced phonological skills.

The methodological and sampling differences of the studies included in the review call for caution in the overall conclusion of our discussion. Furthermore, it is worth addressing that studies with small sample sizes were not excluded from the present review. Although this could potentially reduce the reliability of the findings, we decided to include them, together with old contributions, which are commonly discarded by systematic reviews, to have a comprehensive picture of how research about connections between DD and music developed. Further evidence from methodologically sound study designs is needed to conduct a systematic quantitative review that could further the knowledge about the impact of auditory and musical intervention for reading difficulties.

Many authors suggested that musical interventions should be used as complements to standard remedial methods. Based on the multi-componential nature of both neurobiological (Richlan, 2014) and behavioural manifestations (Menghini et al., 2010) of DD, we argue that interventions centred on music and auditory processing training should include phonological and reading activities as well. Embedding decoding tasks into musical/auditory-based exercises would indeed facilitate the translational beneficial effect on reading performance.

## Declarations

### Author contribution statement

Alice Cancer; Alessandro Antonietti: All authors listed have significantly contributed to the development and the writing of this article.

### Funding statement

This work was supported by the 10.13039/501100005743Università Cattolica del Sacro Cuore of Milan (D.1 2021 funding grant).

### Data availability statement

No data was used for the research described in the article.

### Declaration of interests statement

The authors declare no conflict of interest.

### Additional information

No additional information is available for this paper.

## References

[bib1] American Psychiatric Association (2013).

[bib2] Besson M., Chobert J., Marie C. (2011). Transfer of training between music and speech: common processing, attention, and memory. Front. Psychol..

[bib3] Bhide A., Power A., Goswami U. (2013). A rhythmic musical intervention for poor readers: a comparison of efficacy with a letter-based intervention. Mind, Brain, and Education.

[bib4] Bonacina S., Cancer A., Lanzi P.L., Lorusso M.L., Antonietti A. (2015). Improving reading skills in students with dyslexia: the efficacy of a sublexical training with rhythmic background. Front. Psychol..

[bib5] Butzlaff R.O.N. (2000). Can music be used to teach reading?. J. Aesthetic. Educ..

[bib6] Cancer A., Antonietti A. (2017). Remedial interventions for developmental dyslexia: how neuropsychological evidence can inspire and support a rehabilitation training. Neuropsychol. Trends.

[bib7] Cancer A., Antonietti A. (2018). Rapid automatized naming, verbal working memory, and rhythm discrimination as predictors of reading in Italian undergraduate students with and without dyslexia. Brain Sci..

[bib8] Cancer A., Bonacina S., Antonietti A., Salandi A., Molteni M., Lorusso M.L. (2020). The effectiveness of interventions for developmental dyslexia: rhythmic reading training compared with hemisphere-specific stimulation and action video games. Front. Psychol..

[bib9] Cancer A., Bonacina S., Lorusso M.L., Lanzi P.L., Antonietti A., Serino S., Matic A., Giakoumis D., Lopez G., Cipresso P. (2016). *Pervasive Computing Paradigms For Mental Health* Communications in Computer and Information Science.

[bib10] Cancer A., Sarti D., De Salvatore M., Granocchio E., Chieffo D.P.R., Antonietti A. (2021). Dyslexia telerehabilitation during the COVID-19 pandemic: results of a rhythm-based intervention for reading. Children.

[bib80] Cancer A., De Salvatore M., Granocchio E., Andreoli L., Antonietti A., Sarti D. (2022). The Role of Auditory and Visual Components in Reading Training: No Additional Effect of Synchronized Visual Cue in a Rhythm-Based Intervention for Dyslexia. Appl. Sci..

[bib11] Cancer Stievano, Pace Colombo, Antonietti (2019). Cognitive processes underlying reading improvement during a rhythm-based intervention. A small-scale investigation of Italian children with dyslexia. Children.

[bib12] Cantiani C., Ortiz-Mantilla S., Riva V., Piazza C., Bettoni R., Musacchia G. (2019). Reduced left-lateralized pattern of event-related EEG oscillations in infants at familial risk for language and learning impairment. Neuroimage: Clinic.

[bib13] Cogo-Moreira H., Andriolo R.B., Yazigi L., Ploubidis G.B., de Avila C.R.B., Mari J.J. (2012). Music education for improving reading skills in children and adolescents with dyslexia. Cochrane Database Syst. Rev..

[bib14] Cogo-Moreira H., Brandão de Ávila C.R., Ploubidis G.B., Mari J.D.J. (2013). Effectiveness of music education for the improvement of reading skills and academic achievement in young poor readers: a pragmatic cluster-randomized, controlled clinical trial. PLoS One.

[bib15] Coltheart M., Curtis B., Atkins P., Haller M. (1993). Models of reading aloud: dual-route and parallel-distributed-processing approaches. Psychol. Rev..

[bib16] Coltheart M., Rastle K., Perry C., Langdon R., Ziegler J. (2001). DRC: a dual route cascaded model of visual word recognition and reading aloud. Psychol. Rev..

[bib17] Cunningham J., Nicol T., Zecker S.G., Bradlow A., Kraus N. (2001). Neurobiologic responses to speech in noise in children with learning problems: deficits and strategies for improvement. Clin. Neurophysiol..

[bib18] Cunningham J., Nicol T., Zecker S., Kraus N. (2000). Speech-evoked neurophysiologic responses in children with learning problems: development and behavioral correlates of perception. Ear Hear..

[bib19] Curtin S. (2010). Young infants encode lexical stress in newly encountered words. J. Exp. Child Psychol..

[bib20] Danon-Boileau L., Barbier D. (2002).

[bib21] Diehl S.F. (1999). Listen and learn? A software review of Earobics®. Lang. Speech Hear. Serv. Sch..

[bib22] Douglas S., Willatts P. (1994). The relationship between musical ability and literacy skills. J. Res. Read..

[bib23] Eckert M. (2004). Neuroanatomical markers for Dyslexia: a review of Dyslexia structural imaging studies. Neuroscientist.

[bib24] Flaugnacco E., Lopez L., Terribili C., Montico M., Zoia S., Schön D. (2015). Music training increases phonological awareness and reading skills in Developmental Dyslexia: a randomized control trial. PLoS One.

[bib25] Flaugnacco E., Lopez L., Terribili C., Zoia S., Buda S., Tilli S. (2014). Rhythm perception and production predict reading abilities in developmental dyslexia. Front. Hum. Neurosci..

[bib26] Forgeard M., Schlaug G., Norton A., Rosam C., Iyengar U., Winner E. (2008). The relation between music and phonological processing in normal-reading children and children with dyslexia. Music Percept..

[bib27] Franceschini S., Gori S., Ruffino M., Pedrolli K., Facoetti A. (2012). A causal link between visual spatial attention and reading acquisition. Curr. Biol..

[bib28] Franceschini S., Gori S., Ruffino M., Viola S., Molteni M., Facoetti A. (2013). Action video games make dyslexic children read better. Curr. Biol..

[bib29] Gaab N., Gabrieli J.D.E., Deutsch G.K., Tallal P., Temple E. (2007). Neural correlates of rapid auditory processing are disrupted in children with developmental dyslexia and ameliorated with training: an fMRI study. Restor. Neurol. Neurosci..

[bib30] Giraud A.-L., Ramus F. (2013). Neurogenetics and auditory processing in developmental dyslexia. Curr. Opin. Neurobiol..

[bib31] Gordon R.L., Fehd H.M., McCandliss B.D. (2015). Does music training enhance literacy skills? A meta-analysis. Front. Psychol..

[bib32] Goswami U. (2011). A temporal sampling framework for developmental dyslexia. Trends Cognit. Sci..

[bib33] Goswami U., Fosker T., Huss M., Mead N., Szűcs D. (2011). Rise time and formant transition duration in the discrimination of speech sounds: the Ba-Wa distinction in developmental dyslexia. Dev. Sci..

[bib34] Goswami U., Gerson D., Astruc L. (2010). Amplitude envelope perception, phonology and prosodic sensitivity in children with developmental dyslexia. Read. Writ..

[bib35] Goswami U., Huss M., Mead N., Fosker T., Verney J.P. (2013). Perception of patterns of musical beat distribution in phonological developmental dyslexia: significant longitudinal relations with word reading and reading comprehension. Cortex.

[bib36] Goswami U., Power A.J., Lallier M., Facoetti A. (2014). Oscillatory “temporal sampling” and developmental dyslexia: toward an over-arching theoretical framework. Front. Hum. Neurosci..

[bib37] Habib M., Lardy C., Desiles T., Commeiras C., Chobert J., Besson M. (2016). Music and dyslexia: a new musical training method to improve reading and related disorders. Front. Psychol..

[bib38] Hämäläinen J.A., Salminen H.K., Leppänen P.H.T. (2013). Basic auditory processing deficits in dyslexia: systematic review of the behavioral and event-related potential/field evidence. J. Learn. Disabil..

[bib39] Hayes E.A., Warrier C.M., Nicol T.G., Zecker S.G., Kraus N. (2003). Neural plasticity following auditory training in children with learning problems. Clin. Neurophysiol..

[bib40] Huss M., Verney J.P., Fosker T., Mead N., Goswami U. (2011). Music, rhythm, rise time perception and developmental dyslexia: perception of musical meter predicts reading and phonology. Cortex.

[bib41] Kyle F., Kujala J., Richardson U., Lyytinen H., Goswami U. (2013). Assessing the effectiveness of two theoretically motivated computer-assisted reading interventions in the United Kingdom: GG Rime and GG Phoneme. Read. Res. Q..

[bib42] Lallier M., Molinaro N., Lizarazu M., Bourguignon M., Carreiras M. (2017). Amodal atypical neural oscillatory activity in Dyslexia: a cross-linguistic perspective. Clin. Psychol. Sci..

[bib43] Leong V., Kalashnikova M., Burnham D., Goswami U. (2014). (Singapore).

[bib44] Lorusso M.L., Facoetti A., Paganoni P., Pezzani M., Molteni M. (2006). Effects of visual hemisphere-specific stimulation versus reading-focused training in dyslexic children. Neuropsychol. Rehabil..

[bib45] Magnan A., Ecalle J. (2006). Audio-visual training in children with reading disabilities. Comput. Educ..

[bib46] McArthur G.M., Ellis D., Atkinson C.M., Coltheart M. (2008). Auditory processing deficits in children with reading and language impairments: can they (and should they) be treated?. Cognition.

[bib47] Menghini D., Finzi A., Benassi M., Bolzani R., Facoetti A., Giovagnoli S. (2010). Different underlying neurocognitive deficits in developmental dyslexia: a comparative study. Neuropsychologia.

[bib48] Merzenich M.M., Jenkins W.M., Johnston P., Schreiner C., Miller S.L., Tallal P. (1996). Temporal processing deficits of language-learning impaired children ameliorated by training. Science.

[bib49] Musacchia G., Sams M., Skoe E., Kraus N. (2007). Musicians have enhanced subcortical auditory and audiovisual processing of speech and music. Proc. Natl. Acad. Sci. U. S. A.

[bib50] Overy K. (2003). Dyslexia and music. From timing deficits to musical intervention. Ann. N. Y. Acad. Sci..

[bib51] Ozernov-Palchik O., Wolf M., Patel A.D. (2018). Relationships between early literacy and nonlinguistic rhythmic processes in kindergarteners. J. Exp. Child Psychol..

[bib52] Patel A.D. (2003). Language, music, syntax and the brain. Nat. Neurosci..

[bib53] Patel A.D. (2012). The OPERA hypothesis: assumptions and clarifications. Ann. N. Y. Acad. Sci..

[bib54] Ramus F., Marshall C.R., Rosen S., Lely van der H.K.J. (2013). Phonological deficits in specific language impairment and developmental dyslexia: towards a multidimensional model. Brain.

[bib55] Register D., Darrow A.-A., Swedberg O., Standley J. (2007). The use of music to enhance reading skills of second grade students and students with reading disabilities. J. Music Ther..

[bib56] Richlan F. (2014). Functional neuroanatomy of developmental dyslexia: the role of orthographic depth. Front. Hum. Neurosci..

[bib57] Rolka E.J., Silverman M.J. (2015). A systematic review of music and dyslexia. Arts Psychother..

[bib58] Roskam K. (1979). Music therapy as an aid for increasing auditory awareness and improving reading skill. J. Music Ther..

[bib59] Saine N.L., Lerkkanen M.-K., Ahonen T., Tolvanen A., Lyytinen H. (2011). Computer-assisted remedial reading intervention for school beginners at risk for reading disability. Child Dev..

[bib60] Schön D., Magne C., Besson M. (2004). The music of speech: music training facilitates pitch processing in both music and language. Psychophysiology.

[bib61] Sergeant D., Vhatcher G. (1974). Intelligence, social status and musical abilities. Psychol. Music.

[bib62] Snowling M.J., Hulme C. (2012). Annual Research Review: the nature and classification of reading disorders – a commentary on proposals for DSM-5. JCPP (J. Child Psychol. Psychiatry).

[bib63] Standley J.M. (2008). Does music instruction help children learn to read? Evidence of a meta-analysis. Update Appl. Res. Music Educ..

[bib64] Stein J. (2001). The magnocellular theory of developmental dyslexia. Dyslexia.

[bib65] Strait D.L., O’Connell S., Parbery-Clark A., Kraus N. (2014). Musicians’ enhanced neural differentiation of speech sounds arises early in life: developmental evidence from ages 3 to 30. Cerebr. Cortex.

[bib66] Strehlow U., Haffner J., Bischof J., Gratzka V., Parzer P., Resch F. (2006). Does successful training of temporal processing of sound and phoneme stimuli improve reading and spelling?. Eur. Child Adolesc. Psychiatr..

[bib67] Tallal, Miller S., Fitch R.H. (1993). Neurobiological basis of speech: a case for the preeminence of temporal processing. Ann. N. Y. Acad. Sci..

[bib68] Tallal P. (2004). Improving language and literacy is a matter of time. Nat. Rev. Neurosci..

[bib69] Tallal P., Gaab N. (2006). Dynamic auditory processing, musical experience and language development. Trends Neurosci..

[bib70] Tallal P., Miller S.L., Bedi G., Byma G., Wang X., Nagarajan S.S. (1996). Language comprehension in language-learning impaired children improved with acoustically modified speech. Science.

[bib71] Temple E., Deutsch G.K., Poldrack R. a, Miller S.L., Tallal P., Merzenich M.M. (2003). Neural deficits in children with dyslexia ameliorated by behavioral remediation: evidence from functional MRI. Proc. Natl. Acad. Sci. U. S. A.

[bib72] Temple E., Poldrack R., Protopapas A. al, Nagarajan S., Salz T., Tallal P. (2000). Disruption of the neural response to rapid acoustic stimuli in dyslexia: evidence from functional MRI. Proc. Natl. Acad. Sci. Unit. States Am..

[bib73] Thomson J.M., Goswami U. (2008). Rhythmic processing in children with developmental dyslexia: auditory and motor rhythms link to reading and spelling. J. Physiol. Paris.

[bib74] Thomson J.M., Leong V., Goswami U. (2012). Auditory processing interventions and developmental dyslexia: a comparison of phonemic and rhythmic approaches. Read. Writ..

[bib75] Tierney A., Kraus N. (2013). Music training for the development of reading skills. Prog. Brain Res..

[bib76] Tierney A., Kraus N. (2014). Auditory-motor entrainment and phonological skills: precise auditory timing hypothesis (PATH). Front. Hum. Neurosci..

[bib77] Wible B., Nicol T., Kraus N. (2002). Abnormal neural encoding of repeated speech stimuli in noise in children with learning problems. Clin. Neurophysiol..

[bib78] Woodruff Carr K., White-Schwoch T., Tierney A.T., Strait D.L., Kraus N. (2014). Beat synchronization predicts neural speech encoding and reading readiness in preschoolers. Proc. Natl. Acad. Sci. U. S. A.

[bib79] Ziegler J.C., Perry C., Zorzi M. (2014). Modelling reading development through phonological decoding and self-teaching: implications for dyslexia. Philos. Trans. R. Soc. Lond. B Biol. Sci..

